# Labeling of methyl groups: a streamlined protocol and guidance for the selection of ^2^H precursors based on molecular weight

**DOI:** 10.1007/s10858-024-00441-y

**Published:** 2024-05-24

**Authors:** Alexandra Locke, Kylee Guarino, Gordon S. Rule

**Affiliations:** Department of Biological Sciences, Carnegie Mellon University, 4400 5th Ave, Pittsburgh, PA 15213 USA

**Keywords:** Biotechnology, Biophysics, Bacterial expression, NMR, Protein structure

## Abstract

A streamlined one-day protocol is described to produce isotopically methyl-labeled protein with high levels of deuterium for NMR studies. Using this protocol, the D_2_O and ^2^H-glucose content of the media and protonation level of ILV labeling precursors (ketobutyrate and ketovalerate) were varied. The relaxation rate of the multiple-quantum (MQ) state that is present during the HMQC-TROSY pulse sequence was measured for different labeling schemes and this rate was used to predict upper limits of molecular weights for various labeling schemes. The use of deuterated solvents (D_2_O) or deuterated glucose is not required to obtain ^1^H–^13^C correlated NMR spectra of a 50 kDa homodimeric protein that are suitable for assignment by mutagenesis. High quality spectra of 100–150 kDa proteins, suitable for most applications, can be obtained without the use of deuterated glucose. The proton on the β-position of ketovalerate appears to undergo partial exchange with deuterium under the growth conditions used in this study.

**Supplementary Information:**

The online version contains supplementary material available at 10.1007/s10858-024-00441-y.

## Introduction

Larger biomolecular systems have been accessible to NMR studies by the advent of deuteration, TROSY techniques, and in the case of proteins, methyl-specific labeling. Deuteration reduces spin–spin relaxation, allowing transfer of coherence over longer distances(Sattler and Fesik 1996). TROSY techniques select for the more slowly decaying excited state, enhancing resolution and sensitivity (Pervushin et al. 1997; Tugarinov and Kay 2004). In addition, the presence of three hydrogens on the methyl group, coupled with rapid methyl rotation, further enhances the signal from these groups. Methyl-specific labeling, typically Ile, Val, and Leu (ILV) residues, provides suitable spin probes to measure structure and dynamics at many sites in most proteins (Goto et al. 1999). Additional information can be obtained from methyl labeled Ala (Ayala et al. 2009) and Thr residues (Velyvis et al. 2012). High levels of deuteration of methyl-labeled samples increases sensitivity and resolution by reducing methyl proton and carbon relaxation rates (Goto et al. 1999).

An important requirement for the interpretation of NMR-derived properties, such as structural information, dynamics, and ligand induced chemical shifts, is the assignment of a resonance peak to an individual group in the protein. In the case of methyl resonances, several approaches have been successfully used (Pritišanac et al. 2020; Clay et al. 2022). NMR based approaches include: (i) spin–spin coherence transfer (Tugarinov and Kay 2003), (ii) through-space dipolar coupling (Nerli et al. 2021), (iii) paramagnetic enhancement (PRE) using nitroxides or lanthanides (Pritišanac et al. 2020), and (iv) pseudo contact shifts from lanthanides with anisotropic magnetic susceptibility tensors (John et al. 2007; Lescanne et al. 2017). Coherence transfer methods require mainchain assignments and becomes more difficult to apply for larger proteins (~ 50 kDa). Although dipolar coupling approaches become more efficient in larger systems, they rely on a known structure of the protein and methyl groups that are distant from other methyl groups cannot be assigned using this approach. Paramagnetic enhancement can provide useful assignment information; however, it can be difficult to estimate the position of the unpaired electron on nitroxides due to flexibility of the paramagnetic group. Lanthanide based pseudocontact shifts provide very useful orientation information for assignments beyond PREs but require the creation of a lanthanide binding site.

Another common approach for methyl assignments is site-directed mutagenesis(Amero et al. 2011; Crublet et al. 2014). In this case individual Ile residues are replaced by Val, Val by Ala, and Leu by Ala. Replacement of Ala and Thr residues by Gly and Ser respectively also provide an efficient way to assign methyl resonances from those residues. The mutation causes the signal from the mutated residue to either move to a different region of the spectrum (e.g. Ile to Val) or disappear entirely from the spectrum (e.g. Thr to Ser). This approach is very robust for Ile since the replacement of Ile by Val generally has little effect on protein structure (Trivedi and Nagarajaram 2020) and therefore little effect on the chemical shifts of the remaining Ile residues. This approach can also be very effective for Thr, Val, Leu, and Ala residues with the judicious selection of mutation sites and type of substitution, based on structure-based predictions (e.g. molecular dynamics, MD) of the effect of individual substitutions on the protein structure. For example, although Thr to Ser replacements are conservative, MD calculations may show that replacement of Thr by Val, Ala, or Gly may be less perturbing at some sites.

With the advent of expedient, automatable, and low-cost methods of gene synthesis as well as robust methods for chemical lysis of cells (Listwan et al. 2010), it is practical to generate a large panel of mutants for methyl assignment purposes that would include all Ile residues and a substantial number of Thr, Val, Leu, and Ala residues. Naturally, methyl labeled protein must be generated for each of these mutants. Consequently, it is advantageous to streamline the protein production pipeline and reduce the cost of labeled precursors.

Several approaches have been published that provide workflows to produce deuterated proteins (Crublet et al. 2014; Li and Byrd 2022; Cai et al. 2021, 2016). Although these workflows are reliable, they are somewhat time consuming to produce many samples, often requiring sequential adaptation to different media conditions with centrifugation. We present a more streamlined protocol for the adaptation of cells to deuterated media that allows adaptation and protein induction in one day, with the subsequent harvest of the cells the following day. Centrifugation of the culture during this process is not required unless very high levels (> 97.5%) of D_2_O are required. This streamlined approach facilitates a high-throughput approach to the production of an array of mutant proteins for assignment or other mutational mapping purposes.

An important consideration in the generation of multiple samples is the efficient use of isotopes (D_2_O, deuterated glucose, ILV methyl precursors) that are required to make highly deuterated samples. For example, Clore has shown that high levels of deuterated glucose can be used to obtain very dense cell cultures post-induction, reducing the deuterium requirements because of increased protein yield per volume of culture (Cai et al. 2016). A simple way to reduce isotope costs is to reduce the deuterium content of the solvent (H_2_O versus D_2_O), glucose, and precursors. We have systematically explored the reduction of deuterium content of the solvent, glucose, and ketoacid precursors on the quality of ^1^H–^13^C correlation spectra with the goal to reduce cost by establishing guidelines for the labeling of moderate-to-large sized proteins (e.g. 25–250 kDa). We show that it is possible to obtain 2D ^1^H–^13^C spectra of a 50 kDa protein that are suitable for resonance assignment purposes by growing the cells in the presence of 100% H_2_O and protonated glucose, with the only source of deuterium from highly deuterated ^13^C precursors. As the protein size increases, a higher percentage of D_2_O and deuterated glucose are required. Guidelines are provided to select deuterium levels that are appropriate for different molecular weights. We show that the use of deuterated glucose is not required unless the molecular weight of the protein exceeds ~ 125 kDa; high quality HMQC spectra can be obtained from large proteins that are obtained from cultures grown in 90% D_2_O and 100% ^1^H–glucose.

## Results and discussion

### Streamlined process for cell growth

**Fig. 1:**
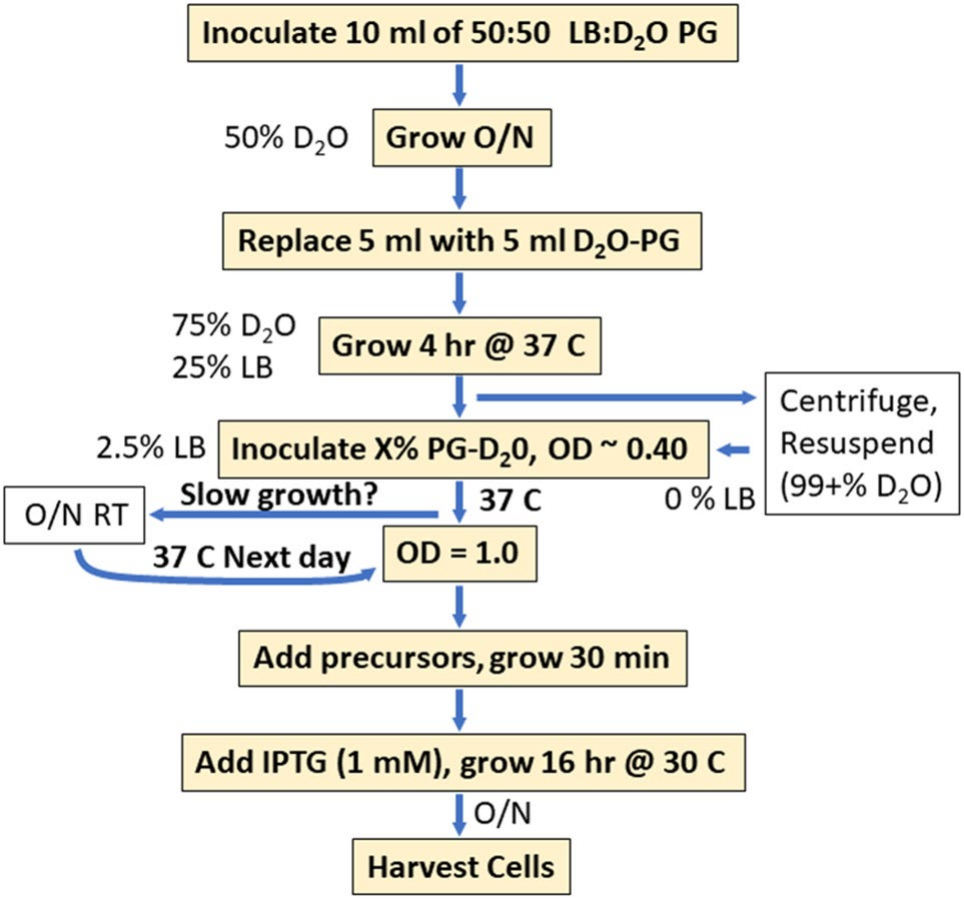
Process for robust growth in high levels of D_2_O. The flow chart indicates the key steps in the production of deuterated protein. The optional path on the right provides a higher level of deuteration but requires centrifugation and results in a longer growth period on the same day. The optional path (open box) on the left is for occasional slow growth that prohibits induction on the same day. *O/N* overnight

A rapid and reliable method of obtaining deuterated protein was developed by combining adaptation with a change in media from rich to minimal media. The overall strategy is shown in Fig. 1. Overnight growth of cells in 50% D_2_O while still in 50% rich media (LB), followed by a short 4 h growth phase at increased D_2_O/decreased LB levels the next morning allows the cells to adapt more quickly to growth at higher D_2_O levels within the same day. The level of deuteration of the protein in the purified protein depends on the deuterium content of the solvent (D_2_O) and whether protonated or deuterated glucose is used (Table 1). Higher levels of deuteration are achieved by increasing the level of D_2_O as well as including deuterated glucose, at the expense of an increased time to protein induction. D_2_O levels as high as 97.5% can be obtained without any centrifugation step. If higher levels of D_2_O are required, it is necessary to centrifuge the cells and resuspend at the required D_2_O level. The overall growth protocol is also suitable for Ala and Thr methyl labeling in combination with ILV labeling.

**Table 1:** Effect of growth conditions on deuteration levels, growth time, and upper limit for molecular weight

Growth condition		^2^H level (%)	Growth time to OD 1 (h)^a^	MW kDa (upper limit)	MQ R_2_ (s^−1^)
D_2_O%	Glucose				
0	^1^H/^2^H 100:0	0%	2.0	61	49.0
25	^1^H/^2^H 75:25	11%	N.D	75	40.0
50	^1^H/^2^H 50:50	30%	N.D	86	34.9
75	^1^H/^2^H 25:75	60%	N.D	102	29.4
90	^1^H/^2^H 10:90	81%	4.0	154	19.5
90^b^	^1^H/^2^H 10:90(LB)	81%	4.0	146	20.5
90	^1^H 100%	71%	3.7	132	22.7
99.0^c^	^2^H	96%	5.7	192	15.6
97.5^d^	^2^H (LB)	93%	4.7	186	16.1
99.0^c^	^1^H	83%	5.3	162	18.5

All samples were labeled using ketobuturate that was deuterated on the β-position and ketovalerate that was deuterated on the β-position and on one of the methyl groups

^a^Assuming starting OD = 0.4

^b^2.5% LB remaining (no centrifugation)

^c^Centrifugation required to attain level of D_2_O content

^d^2.5% LB remaining, highest D_2_O level without centrifugation

### Effect of solvent and glucose deuterium levels

Table 1 shows the effect of D_2_O level and protonation/deuteration state of glucose on the level of incorporated deuterium, growth time, and the MQ relaxation rate. The MQ relaxation rate was used as a proxy for overall sensitivity. This rate was measured when the magnetization for both spins was transverse, i.e. essentially during the carbon evolution period (see Fig. S3). This permitted the direct measurement of the effect of surrounding protons and deuterons on the relaxation rate of the ^13^CH_3_ methyl group. Analysis of the relaxation of CH_3_ groups during a HMQC sequence in large proteins by Ollerenshaw et al. (2003) showed that the signal intensity is:

$$I \propto e^{{\left( { - \left[ {4\tau + t_{2} } \right]R_{2,H }^{s} - t_{1} R_{2,CH}^{s} } \right)}} .$$

The proton relaxation rate, ${R}_{2,H}^{S}$ = R_CH_ + R_ext_, where R_CH_ is the relaxation rate due to intra-methyl proton-carbon dipolar coupling and R_ext_ is the rate due to coupling to surrounding protons and deuterons. In contrast, ${R}_{2,CH}^{S}$ (= MQ relaxation rate) only reflects relaxation from adjacent protons and deuterons due to complete cancelation of intra-methyl relaxation due to the TROSY effect (Ollerenshaw et al. 2003). Consequently, ${R}_{2,CH}^{S}$ is more reflective of the effect of residual protons on the overall sensitivity of the experiment because it directly determines the linewidth in ω_1_ and it contributes to the linewidth in ω_2_ and signal loss during magnetization transfer (4τ period), via R_ext_.

**Fig. 2:**
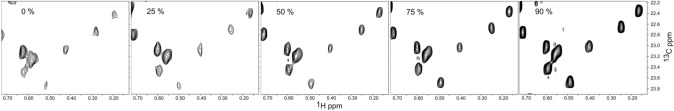
Effect of deuteration level on the quality of the HMQC ILV spectra. A region of the spectra containing Leu/Val peaks is shown for proteins purified after expression in the indicated D_2_O concentrations

We investigated how varying the deuterium level of both D_2_O and glucose, from 0 to 90% (rows 1–5, Table 1), affected the quality of the spectra (Fig. 2) and the MQ relaxation rate. Figures 2, 7 and S4 show that samples grown in 0% D_2_O and protonated glucose give spectra with adequate resolution and sensitivity to easily detect all the peaks in the spectra of the 50 kDa A1-1 protein used in this study. The sensitivity of detection could be improved by acquiring more scans over a shorter carbon evolution period or using SOFAST techniques(Schanda et al. 2005), but these approaches were not explored here. Increasing the D_2_O content and ^2^H-glucose content increases the quality of the spectra (Fig. 2), as well as decreasing the MQ relaxation rate (Table 1), as expected.

The level of sensitivity and resolution that is obtained with growth in 100% H_2_O is because the heavily deuterated precursors that were used place deuterium atoms in favorable locations to reduce spin–spin coupling to the methyl protons. The hydrogens on the same residue are among those closest to the labeled methyl and these have been replaced by deuterons. In addition, the hemi-deuterated methyl groups on Val and Leu reduce the overall proton density within the core of the protein. Calculations based on local proton density indicate that deuteration of the γ_1_-protons on Ile reduce the relaxation rate of the Ile δ methyl by approximately 30%. In the case of Val and Leu residues, the deuteration of the β/γ proton reduces the relaxation rate by 15% and deuteration of one methyl reduces the rate by an additional 30%. This analysis is consistent with results presented by Dubey et al. (2021) who showed that the local deuteration of leucine reduces the relaxation rate of leucine methyl groups in proteins. The principal application of Dubey’s approach is for labeling with yeast and mammalian cells expression systems. The work presented here indicates that the synthesis of similarly labeled Val and Ile would also be advantageous in those systems.

The MQ-relaxation rates that were obtained under different labeling schemes were used to predict the approximate upper molecular weight limit for that scheme (see Fig. 8). We used a heuristic approach to relate the MQ relaxation rate for a particular labeling scheme to a suggested molecular weight limit. We assumed an average MQ-relaxation rate of ~ 60 s^−1^ would give spectra of suitable sensitivity and dispersion. This value was chosen because the relaxation rate for ILV resonances in A1-1 grown with ^1^H glucose and H_2_O showed a median relaxation rate of 49 s^−1^ for most residues while weak resonances in that sample had relaxation rates > 70 s^−1^.

To convert MQ relaxation rates to effective molecular weight we associated the τ_c_ for A1-1 in D_2_O at 300 K (~ 30 ns), with the observed relaxation rate (R) for a particular labeling scheme. The τ_c_ that would result in a rate of 60 s^−1^ under those conditions was simply 30 ns × 60 s^−1^/R. The τ_c_ was converted to molecular weight by dividing by 0.6 (Rossi et al. 2010). For example, the sample grown in ^1^H glucose and H_2_O gave a relaxation rate of 49 s^−1^ for A1-1. Therefore, a relaxation rate of 60 s^−1^ corresponds to a τ_c_ of 36.7 ns (= 30 ns × 60/49), corresponding to a MW of 61 kDa. The slowest relaxation rate we measured, with growth in 99% D_2_O and ^2^H-glucose was 15.6 s^−1^. The τ_c_ to give a rate of 60 s^−1^ under that labeling condition is 30 × 60/15.6 = 115 ns, corresponding to a MW of 192 kDa.

**Fig. 3:**
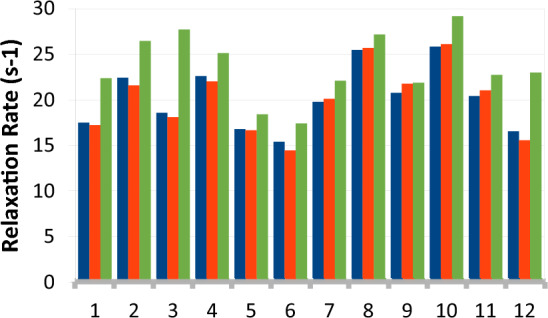
Effect of Residual LB and Glucose source on MQ relaxation rates for individual Ile residues. Cells were grown in 90% D_2_O in the presence of 90% ^2^H-glucose (blue, first bar), with residual 2.5% LB (red, second bar), or with 90% D_2_O and 100%.^1^H-glucose (green, third bar)

The process outlined in Fig. 1 will only require a centrifugation step if high levels of D_2_O are required. We found that the residual LB media (2.5%) during the growth and induction phase has little effect on the deuterium level in the sample as well as the MQ relaxation rate. Figure 3 shows the MQ relaxation rates for Ile residues, without and with LB (first two bars for each residue). The relaxation rates of individual methyl groups are essentially the same for both conditions. A similar trend is also seen in 99% D_2_O and for Val & Leu residues at both D_2_O levels (not shown).

**Fig. 4:**
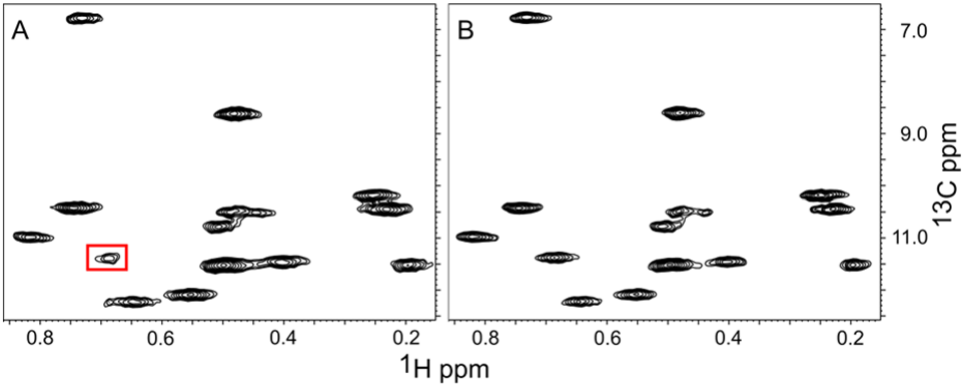
Effect of Glucose Deuteration on Quality of spectra. Cells were grown in 90% D_2_O in the presence of 100% ^1^H-glucose (**A**) or 90%^2^H-glucose (**B**). Spectra were measured in the presence of 20% sucrose at 285 K to increase the apparent molecular weight to ~ 150 kDa. The boxed resonance in panel A is from an Ile that relaxes faster due to a higher local ^1^H density. Regardless, it is clearly observable in the spectra. Otherwise, the spectra and relaxation rates are similar

We also determined which source of deuterons (D_2_O or glucose) had a larger impact on the quality of the spectra and found that D_2_O was more important. The replacement of ^2^H-glucose with ^1^H-glucose at 90% and 99% D_2_O levels leads to minor increases in the MQ relaxation rate (~ 10%, Fig. 3, last bar of each group) and consequently minor decreases in the upper limit for molecular weight (see Table 1). For example, growth in 90% D_2_O and ^1^H-glucose should give acceptable spectra for proteins in the range of ~ 125 kDa. To demonstrate that this is the case we acquired HMQC spectra of A-11 in the presence of 20% sucrose at 285 K. These conditions should increase the rotational correlation time from ~ 28 ns to ~ 90 ns, increasing the apparent molecular weight to ~ 150 kDa. Figure 4 shows the Ile region of the HMQC spectra for samples grown in 90% D_2_O in ^1^H-glucose or ^2^H-glucose. The spectra show similar resolution and peak intensity. One Ile residue shows reduced intensity in the ^1^H-glucose sample due to more rapid relaxation from a higher-than-average local proton density, but it is still clearly visible in the spectra.

The median MQ relaxation rates for the sample grown in ^1^H-glucose were 41 s^−1^ and 46 s^−1^ for Val/Leu and Ile, respectively. Growth in ^2^H-glucose gave MQ relaxation rates of 36 s^−1^ and 39 s^−1^ for Val/Leu and Ile, respectively. The weaker peak in the sample obtained using ^1^H-glucose (boxed in Fig. 4A) had a relaxation rate of 67 s^−1^, suggesting that it may be difficult to obtain signals from all groups using ^1^H-glucose (+ 90% D_2_O) when labeling a 150 kDa protein. This conclusion is consistent with our suggested upper limit of 132 kDa that was predicted from a relaxation rate of 22.7 s^−1^ measured in D_2_O (no glycerol) at 300 K (see Table 1).

**Fig. 5:**
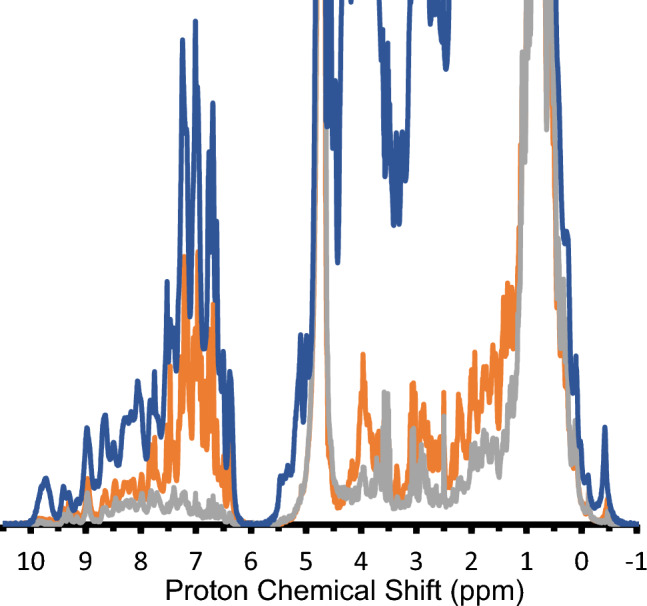
Effect of Glucose Deuteration on Aromatic and Aliphatic Labeling. Cells were grown in 100% H_2_O ^1^H-glucose (blue), 99% D_2_O with either ^1^H glucose (orange) or.^2^H-glucose (gray)

Growth in 100% ^1^H-glucose increased the overall protonation level in the sample but showed a smaller than expected increase in the MQ relaxation rate (see Table 1). The additional protons from ^1^H-glucose appear to be selectively incorporated into aromatic sites as opposed to aliphatic sites. Figure 5 shows the 1D-^1^H spectra of samples grown in H_2_O, 99% D_2_O + ^1^H-glucose, 99% D_2_O with ^2^H-glucose. Selective incorporation into aromatics is a consequence of the pathways for biosynthesis which utilize precursors derived directly from glucose via the pentose pathway while aliphatic residues would use glucose-derived carbons that have undergone H–D exchange during central metabolism (Rosen et al. 1996). The relatively small increase in MQ-relaxation rate for the ^1^H-glucose derived samples may be due to rapid ring flips of Phe and Tyr residues which would reduce dipolar coupling between those aromatics and ILV methyl groups.

### Effect of deuterium labeling of ILV precursors

Ketobutyrate and ketovalerate precursors are commercially available with different levels of deuteration (See Scheme 1, supplementary material). The β-hydrogens on ketobutryate can be protons (HKB) or deuterons (DKB). Ketovalerate can be purchased with both methyls labeled and either a proton (HKV2) or a deuteron at the β-position (DKV2). In addition, ketovalerate with a single ^13^C labeled methyl is available, with all other hydrogens deuterated (KV). We generated protein samples from growth in either 100% H_2_O + 100% ^1^H-glucose, or 90% D_2_O + 90% ^2^H-Glucose, or 90% D_2_O + 100% ^1^H-Glucose, varying the Ile and Leu/Val precursor (e.g. HKB + HKV2, HKB + DKV2, HKB + KV, DKB + HKV2, DKB + DKV2, DKB + KV).

A concern with the use of ketoacid precursors for labeling is chemical exchange of the hydrogens on the acidic C_β_ position on the precursor, e.g. HKB becomes DKB when used with high levels of D_2_O in the media. We see no evidence of proton-deuterium exchange for HKB during growth, based on deuterium isotope shifts (see Fig. S4). The ^2^H isotope shift is − 0.18 ppm (^13^C) from HKB to DKB for samples grown in D_2_O. This is consistent with the reported two-bond shift of -0.09 ppm per deuteron (Maltsev et al. 2012), suggesting that the CH_2_ group at the Cγ position on Ile remains protonated when HKB is used in D_2_O. When DKB is used in conjunction with H_2_O/^1^H-glucose we find a small isotope shift -0.05 ppm. This is presumably a three-bond effect due to the incorporation of a proton at C_β_ during the synthesis of Ile from KB. It is important to note that the source of these protons is from the solvent, not glucose. Growth in D_2_O + ^1^H-glucose does not produce an isotope shift.

**Fig. 6:**
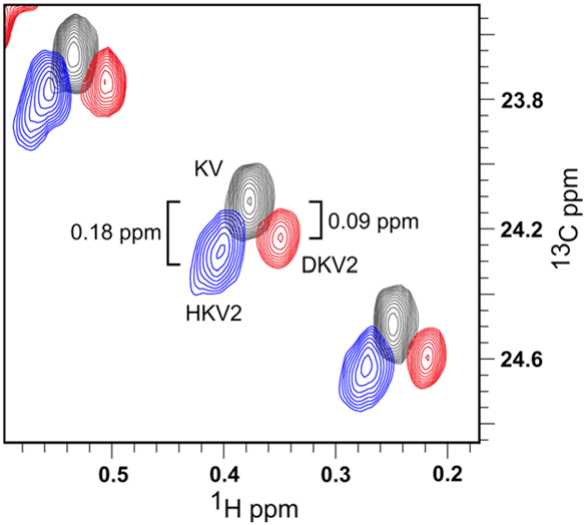
Deuterium-proton exchange of HKV2. Spectra obtained from cells grown in 90% D_2_O + 90% ^2^H glucose in the presence of DKB using different ketovalerate sources. Methyl signals from cells grown in HKV2 show the presence of two overlapping resonances, the lower shifted 0.18 ppm from peaks in the sample grown in KV. The peaks have been displaced laterally to make it easier to see the lineshapes

In the case of ketovalerate labeling (HKV2, DKV2, KV) there appears to be partial exchange of the proton on the β position of HKV2 for deuterium when cells are grown in D_2_O. Figure 6 shows an overlay of spectra obtained from samples grown with different ketovalerate precursors in D_2_O. The resonances obtained from HKV2 labeled samples consist of two overlapping peaks. The lower peak corresponds to isotope shift of + 0.18 ppm relative to the KV peak, which is consistent with 3 deuterons 3 bonds away, and one deuteron that is two bonds away. The upper HKV2 peak has the same shift as the peak for the DKV2 sample, suggesting that the proton at the Cβ position of HKV2 can exchange for a deuteron. The level of exchange is approximately ~ 50%, based on the intensity of each peak. The exchange of H for D in HKV2 also occurs when cells are grown in 90% D_2_O and 100% ^1^H-glucose, indicating that the source of deuterons is the solvent. The β-deuteron on DKV2 and KV appear to be resistant to exchange with protons in H_2_O since the signals in the DKV2 and KV samples are single peaks and the deuterium isotope shift from HKV2 to KV is 0.18 ppm (not shown). The higher exchange rate of the β-proton versus the β-deuteron is consistent with the higher stability of C-D bonds (Wiberg 1955; Martino et al. 2023).

Predicted and measured relaxation rates indicate that the relaxation of methyls on Leu and Val are not greatly affected by protonation or deuteration at the γ-position of Ile. Likewise, the protonation/deuteration state of the β/γ-position on Leu and Val does not have a large effect on the relaxation of the Ile C_δ_ methyl. When only one of the methyls of Leu and Val is labeled (KV precursor), there is a modest reduction in the rate of relaxation of Ile C_δ_ methyls. In the case of DKB labeling in H_2_O, the average relaxation rate of Ile C_δ_ was 52.5 s^−1^ versus 51.1 s^−1^ for DKV2 versus KV, respectively. In the case of DKB labeling in 90% D_2_O, the average relaxation rate of Ile C_δ_ was 20.4 s^−1^ versus 19.2 s^−1^ for DKV2 versus KV, respectively. Since the change in relaxation rates between DKV2 and KV were small (~ 5%) we considered each type of labeling (Ile versus Leu/Val) separately when evaluating relaxation rates.

**Fig. 7:**
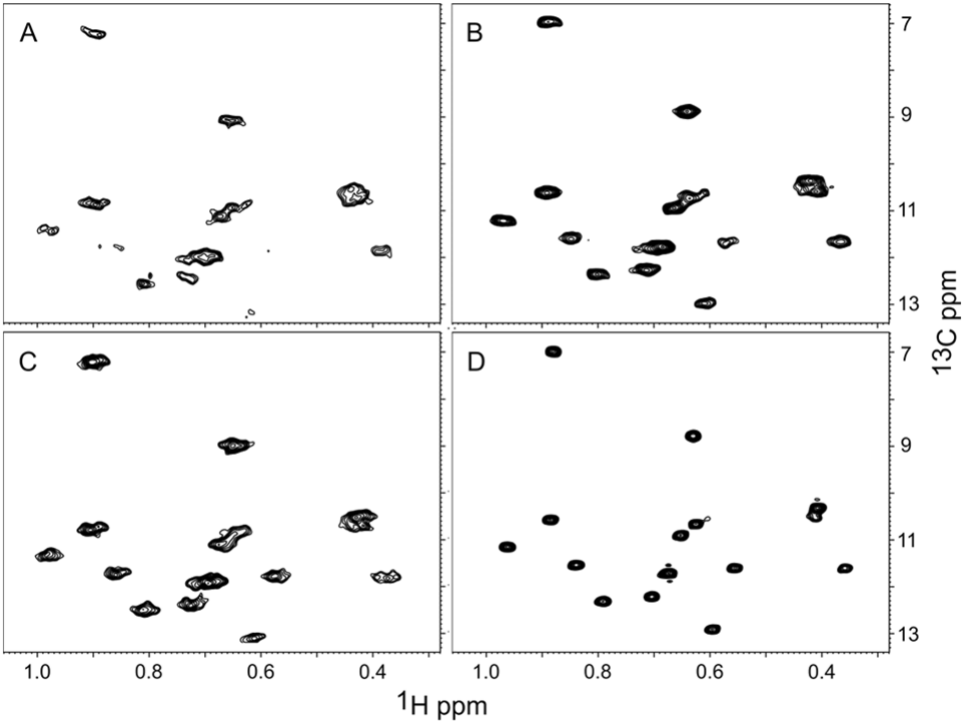
Effect of solvent and protonation of Ketobutyrate on Quality of Ile Spectra. Cells were grown in H_2_O + HKB (**A**), H_2_O + DKB (**B**), 90% D_2_O + HKB (**C**), 90% D_2_0 + DKB (**D**). The quality of the spectra aligns with the MQ relaxation rates: 113 s^−1^ (**A**), 40 s^−1^ (**B**), 67 s^−1^ (**C**), 19 s.^−1^ (**D**)

Table 2 gives the measured relaxation rates and estimated upper limit of molecular weight for combinations of growth media (H_2_O versus 90% D_2_O) and precursor. Note that the relaxation rates given for HKV2 in D_2_O are the average of HKV2 and DKV2 due to partial exchange of the proton with deuterium on HKV2. Figure 7 shows the Ile region of spectra obtained under the four KB labeling schemes presented in Table 2. The quality of the Ile spectrum mirrors the relaxation rate, e.g. HKB + H_2_O <  < HKB + D_2_O ≈ DKB + H_2_O <  < DKB + D_2_O. The quality of Leu + Val spectra (Fig. S2) also show a similar trend.

**Table 2:** Effect of deuteration pattern on relaxation rate, upper limit for molecular weight

Growth Media	HKB		DKB		HKV2		DKV2		KV	
	R2 (s^−1^)	MW	R2 (s^−1^)	MW	R2 (s^−1^)	MW	R2 (s^−1^)	MW	R2 (s^−1^)	MW
H_2_O	113	27 kDa	49	61 kDa	99	30 kDa	67	45 kDa	49	61 kDa
D_2_O (90%)	67	45KDa	19	158 kDa	58	52 kDa	43	70 kDa	19	158 kDa

All samples were labeled with both a ketobutyrate and a ketovalerate precursor. Deuterated ketobutyrate (DKB) was used for all of the ketovalerate data in this table. The R2 rates refer to Ile peaks for ketobutyrate entries and to Val and Leu peaks for HKV2, DKV2, KV entries. HKB has the β-hydrogens protonated, DKB has the β-hydrogens deuterated. HKV2 has both methyls labeled and the β-hydrogen protonated. DKV2 has both methyl groups labeled and the β-hydrogen deuterated. KV has only one methyl group labeled; all remaining hydrogens are deuterated

**Fig. 8:**
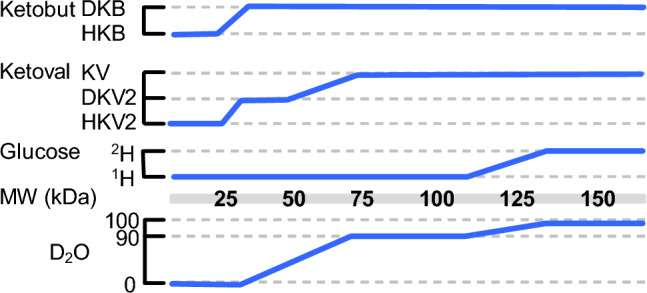
Guidelines for ILV labeling conditions to obtain HSQC spectra suitable for assignments, relaxation dispersion and other uses that do not depend on very high levels of deuteration. The sloped line segments indicate that the choice of deuterated compound may be affected by the nature of the specific protein. For example, a 50 kDa protein may require growth in 90% D_2_O to generate adequate spectra

The different relaxation rates that are obtained for different D_2_O content, glucose labeling, and ketobutyrate and ketovalerate precursors suggest guidelines for isotopic labeling requirements as a function of MW of the protein (see Fig. 8). For example, the suggested labeling scheme for a protein with a molecular weight of 100 kDa should be 90% D_2_O, ^1^H-Glucose, KV and DKB.

Growth on intermediate levels of D_2_O has the potential to generate inhomogeneous line broadening due to a distribution of protons and deuterons on a residue that occurs when the ketoacid precursors are converted to amino acids. For example, the β-hydrogens on Leu will be protons in H_2_O media and deuterons in D_2_O media. In a 50:50 mixed H_2_O:D_2_O media the β-hydrogens will either be two protons, a proton and a deuteron, or two deuterons, with a distribution of 1:2:1. Since the three-bond isotopic shift is small (~ 0.03 ppm) (Maltsev et al. 2012), only a modest increase in linewidth is expected. This increase in linewidth is outweighed by a decrease in linewidth and increase in signal intensity due to higher levels of deuteration (see Fig. 1). Nevertheless, it may be advisable to express the protein in either 100% H_2_O or elevated levels of D_2_O (e.g. 90%), to reduce this effect. 100% H_2_O should be used when labeling with HKV2. Growth in high levels of D_2_O would lead to partial replacement of the β-proton with deuterium, resulting in a decrease in the quality of the spectra (see Fig. 6).

### Alanine and threonine labeling

**Fig. 9:**
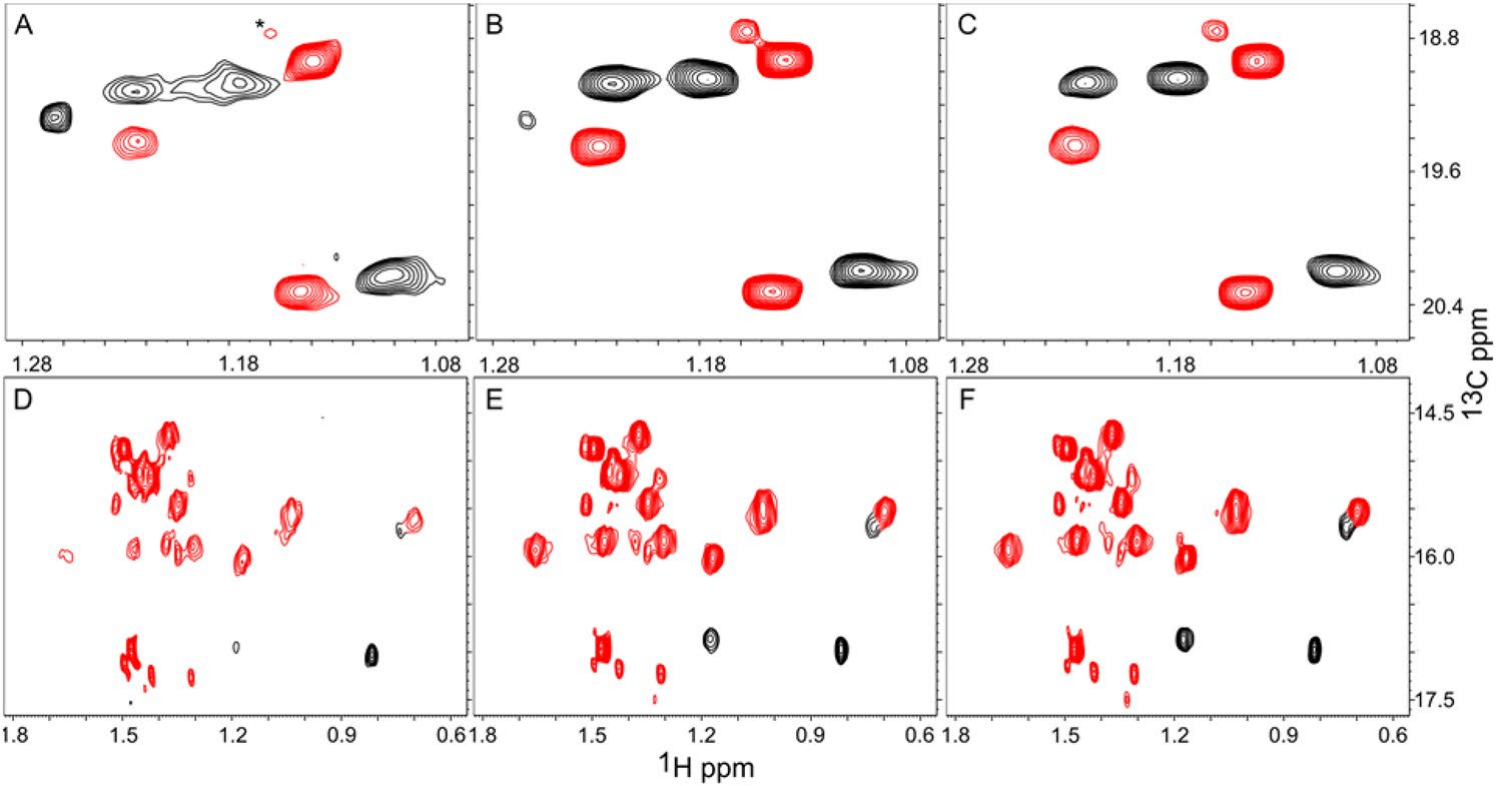
Thr/Ala Methyl Spectra. Thr/Ala peaks are colored red. Thr samples are the upper 3 panels. Cells were grown in H_2_O media (**A**, **D**), 90% D_2_O + 100% ^1^H-glucose (**B**, **E**), 90% (D_2_O + ^2^H-glucose) (**C**, **F**). All samples were grown with DKB and KV. The weaker Thr peak, marked with an * in panel A, is adjacent to only Ile, Leu, and Val residues, causing a greater signal loss

**Fig. 10:**
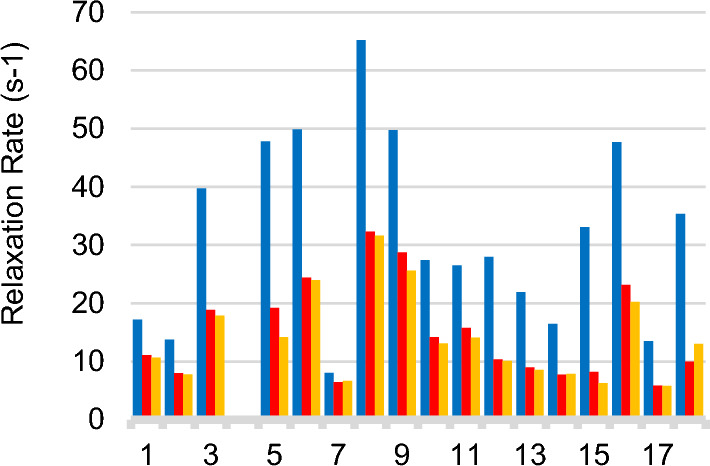
Thr/Ala Relaxation Rates. Groups 1–3 correspond to the three strongest Thr signals in Fig. 9. Groups 5–18 correspond to Ala methyls. First bar in each group (blue) gives the relaxation rate for H_2_O media, the second bar (red) gives the rate for 90% D_2_O + 100% ^1^H-glucose, the third bar gives the rate for 90% D_2_O + 90% ^2^H-glucose

The protein production protocol outlined in Fig. 1 can also be used for Ala + ILV and Thr + ILV labeling. Figure 9 shows spectra of Thr and Ala samples obtained in either 100% H_2_O/^1^H-Glucose, 90% D_2_O + 100% ^1^H-glucose, or in 90% D_2_O + 90% ^2^H-glucose. High quality spectra of Thr methyl groups are obtained under all three conditions. In the case of Ala labeling, growth in H_2_O results in weak signals for some residues because of their faster MQ relaxation rate. Growth in 90% D_2_O with ^1^H-glucose produces spectra that are similar to the spectrum obtained in 90% D_2_O + ^2^H-glucose. This similarity is reflected in the similar MQ relaxation rates shown in Fig. 10. As with ILV labeling, the use of ^2^H-glucose is an unnecessary expense for Thr/Ala labeling for proteins that are smaller than 125 kDa.

Calculations of the MQ relaxation rate for Thr and Ala methyl groups show that a subset of methyl groups can show very high MQ relaxation rates because of nearby ILV methyl groups. This effect is seen for a Thr residue in A1-1 (Fig. 9). Consequently, Ala and Thr spectra with more uniform relaxation rates will be obtained if ILV residues are fully deuterated.

In summary, we present a simple method to obtain methyl labeled deuterated protein that does not require complicated media changes and no centrifugation steps, provided the required D_2_O levels is less than 97.5%. A single centrifugation step is required for higher levels of deuteration. The labeling protocol requires 8–10 h of growth after overnight adaptation, with longer growth periods required for higher levels of deuteration. The protocol is suitable for ILV, Thr, and Ala labeling. MQ relaxation measurements under different deuterium labeling conditions provide guidelines to assist in determining the optimal labeling strategy for different sized protein. For proteins ~ 50 kDa spectra that are adequate for resonance assignment purposes can be obtained by growth in H_2_O with highly deuterated ketobutyrate and ketovalerate precursors. Increasingly higher quality spectra are obtained by increasing the D_2_O content of the media. Deuterons that are provided by the solvent (D_2_O) have a much larger effect on reducing the relaxation rate of the methyls than deuterons from ^2^H-glucose, suggesting that there is little benefit in using ^2^H-glucose for proteins in the 50–125 kDa range.

### Experimental procedures

#### Cell growth

The following process was used to grow up to 100 ml of culture. The recipe for defined media (PG) (Studier 2005) is given in the supplemental material. H_2_O-PG media was made with H_2_O and 5 g protonated glucose/L. D_2_O-PG media was made with 99% D_2_O and 5 gm 99% deuterated glucose/L. Ampicillin was used for selection at 75 mg/L. Cell density was measured using scattering at 600 nm (OD_600_). pET based expression plasmid was transformed into *E. coli* C3013 (New England Biolabs) on Luria broth (LB) plates. After overnight (O/N) growth five to 10 colonies were used to inoculate 10 ml of 50:50 LB:PG-D_2_O (50% D_2_O) and this culture was grown O/N at 37 °C. The next day 5 ml of the culture was replaced with PG-D_2_O to give 75% D_2_O and the culture grown for 4 h at 37 °C. For the purposes of varying the D_2_O content, the cells were centrifuged and resuspended in PG media with various concentrations of D_2_O (0%, 25%, 50%, 75%, 90%, 99%) as well as protonated or deuterated glucose at a cell density of ~ 0.4. The cells were grown at 37 °C until the optical density reached 1.0. At this point precursors for ILV methyl labeling (e.g. 50 mg/L ketobutrate, 100 mg/L ketovalerate) were added (Goto et al. 1999). The ILV methyl-precursors in this study varied in their deuteration level (see Scheme S1 for labeling pattern) to assess the effect of deuteration of the precursors on the quality of the spectra. Unless otherwise noted, the most highly deuterated forms of the keto acids were used. Labeling of Ala + ILV methyls followed the method described by Ayala et al. (2009), except that precursors for ILV labeling were also added as described in Godoy-Ruiz et al. (2010). Note that it is important to adjust the pH of the d-succinic acid to 7.0 before addition to the media. Labeling of ILV + Thr methyls followed the method described by Velyvis et al. (2012). In the case of Ala and Thr labeling, we tested growth in 100% H_2_O, growth in 90% D_2_O with protonated glucose, and in 90% D_2_O with 90% deuterated glucose. All cell growth was performed in baffle flasks with a culture volume that was 10–20% of the flask size.

After addition of the precursors the cells were incubated for 30–45 min, isopropyl thiogalactoside (IPTG) was added to a final concentration of 1 mM and the cells were incubated at 30 °C for 16 h, harvested, and the cell pellet was frozen at -20 °C. The final cell density was ~ 5.0. Occasionally, we experienced slower growth in the 90% D_2_O media such that it was not possible to induce the cells within 4–5 h after the adaptation period. In this case we simply grew the cells O/N at 30 °C, shifted the temperature back to 37 oC, and induced them at OD_600_ = 1.

We also explored the removal of the centrifugation step to streamline cell growth. In this case the cells were simply diluted into the media after the 4-h growth period in 75% D_2_O. The final concentration of LB during labeling is 2.5% and it is possible to generate D_2_O levels as high as 97.5% using this approach. Higher levels of D_2_O require centrifugation and resuspension at the required D_2_O level.

#### Enzyme expression and purification

A synthetic glutathione transferase A1-1 gene was constructed using codon frequencies as described in Boël et al. (2016) using a modified pET vector that contained the complete T7 promoter, as described in Shilling et al. (2020). Previous expression using human codons and tac-based expression vector (Stenberg et al. 1992) gave 55 mg/L. The use of optimized codons and use of the complete T7 promoter increased the yield to 400 mg/L. For purification the cell pellet was resuspended in 8 ml 20 mM Tris, 1 mM EDTA (pH 7.8). Lysozyme was added to a concentration of 1 mg/ml. The cells were frozen in liquid nitrogen, thawed in a RT water bath, and 2 ml of 0.5% triton X-100 was added (final triton 0.1%). Cells were incubated for 15 min on ice. MgSO_4_ was added to 2 mM and 500 units of benzonase was added to digest DNA to reduce viscosity (15 min). The lysate was centrifuged at 30,000 rpm for 20 min (Ti70 rotor). The cleared lysate was loaded on a CM sepharose column, washed, and the protein eluted using a gradient from 0 to 1 M NaCl. Protein yields were approximately 40 mg/100 ml of culture.

The purified protein was concentrated and exchanged into 10 mM Phosphate, 50 mM NaCl, 1 mM EDTA, 0.02% Azide, 100% D_2_O, pH = 7.0, except for the ^15^N-labeled sample which was exchanged into 10% D_2_O. Typical protein concentrations were 500–900 μM.

#### NMR spectroscopy

Spectra were acquired on a Bruker 500 MHz Neo using a 2-channel inverse cryoprobe (Prodigy) using a standard HMQC sequence with echo-antiecho selection for quadrature detection in carbon (Fig. S3A). The total evolution time in carbon was 25.5 ms (80 complex points). Data was processed and visualized using TopSpin NMR (Bruker). Deuteration levels were obtained by integrating a region of the proton spectra (-0.3 to -0.7 ppm) that does not contain signals from ILV, Thr, or Ala methyl groups and normalizing the integral by the concentration of the protein in the sample.

#### Measurement of MQ relaxation rate

The HMQC pulse sequence was modified to include an additional relaxation delay during the coherence selection and t_1_ evolution period of the sequence (see Fig. S3B). Interleaved spectra were collected with different relaxation delay times. Individual spectra were generated using NMRPipe (Delaglio et al. 1995) and the relaxation rate constants were obtained from exponential fitting using routines in NMRPipe.

#### Estimation of rotational correlation time (τ_c_) and upper limit for molecular weight

The rotational correlation time of A1-1 at 300 K was estimated by measuring the rates of decay of the ^15^N α and β spin states in ^15^N labeled deuterated protein using [^15^N,^1^H]-TRACT (Lee et al. 2006)(See Fig. S5 for decay curves). The difference in the relaxation rates is consistent with a τ_c_ of at least 20.5 ns in H_2_O or at least 25 ns after adjustment for the increased viscosity of D_2_O (Millero et al. 1971). Measurement of the ^15^N-T_2_ (500 MHz) using standard CPMG pulse sequences gave a median relaxation time of 28.8 ms, which is consistent with the average correlation time of ~ 27 ns predicted by hydrodynamic calculations (median predicted T_2_ is 28.6 ms by hydroNMR (García de la Torre et al. 2000)). For purposes of estimating molecular weights, we assumed that the correlation time of A1-1 in D_2_O was 30 ns. Higher molecular weights were mimicked by adding sucrose (20%) and the increase in molecular weight was estimated from the increased viscosity(Telis et al. 2007).

#### Calculation of relaxation rates

Relaxation rates of methyl protons were estimated from determining the proton density surrounding the relevant methyl groups. The proton density was adjusted to reflect the growth conditions (D_2_O versus H_2_O), the labeling pattern (e.g. γ-protons or γ -deuterons on Ile residues), and the D_2_O content of the NMR buffer. In the case of the latter, all exchangeable protons were replaced by deuterons.

## Supplementary Information

Below is the link to the electronic supplementary material.

Supplementary file1 (PDF 404 kb)

## Data Availability

All data is described in the manuscript.
