# Supplementary material for: Labeling of methyl groups: a streamlined protocol and guidance for the selection of 2H precursors based on molecular weight
Source: J Biomol NMR. 2024 May 24;78(3):149–59. doi: 10.1007/s10858-024-00441-y (PMC11491418; doi:10.1007/s10858-024-00441-y)
Supplement: Supplementary file 1 — Supplementary file1 (PDF 404 kb) [file 10858_2024_441_MOESM1_ESM.pdf]

Supplemental Material

Labeling of Methyl Groups – A Streamlined Protocol and Guidance for the Selection of ^2H Precursors based on Molecular Weight

Alexandra Locke, Kylee Guarino, Gordon S. Rule*
Department of Biological Sciences, Carnegie Mellon University
4400 5th Ave, Pittsburgh, PA, 15213

*Corresponding author, rule@andrew.cmu.edu

Table S1: PG-Media

Trace elements:

Metal A: $FeCl_3$ solution - 100 mM in 0.12 M HCl (1/100 dilution of conc. HCl)
Metal B: 0.1 M $MnCl_2$ + 0.1 M $ZnSO_4$ in H_2O
Metal C: 20 mM $CoCl_2$, $CuSO_4$, $NiCl_2$, Na_2MoO_4, H_3BO_3, Na_2SeO_3 (can be omitted), in 0.12M HCl.
1000 X stock solution (10 ml), no need to sterilize, store at 4C in dark.

1. 0.0294 gm $CaCl_2$ (hydrate, MW 147 g/m) in 3 ml of H_2O
2. 5 ml of Metal A
3. 1 ml of metal B
4. 1 ml of metal C

Recipe

**Ingredient**	**250 ml**	**300 ml**	**500 ml**	**1 L**
Na_2HPO_4 (50 mM) [FW 141.96]	1.77 g	2.13 g	3.55 g	7.1 g
KH_2PO_4 (50 mM) [FW 136.09]	1.70 g	2.04 g	3.40 g	6.8 g
$(NH_4)_2SO_4$ (25 mM) [132.14]	0.826 g	1.00 g	1.65 g	3.3 g
Glucose [This should be deuterated for D_2O media]	1.25 g	1.50 g	2.5 g	5.0 g
**Mix above, filter sterilize then add:**				
1 M $MgSO_4$ [Sterile stock via filter sterilization] (2 mM final)	0.5 ml	0.6 ml	1.0 ml	2.0 ml
Trace metals 1000X	50 ul	60 ul	0.1 ml	0.2 ml
Thiamine (B1) (1%) [Filter sterilize] Not required for NEB 3013	50 ul	60 ul	0.1 ml	0.2 ml

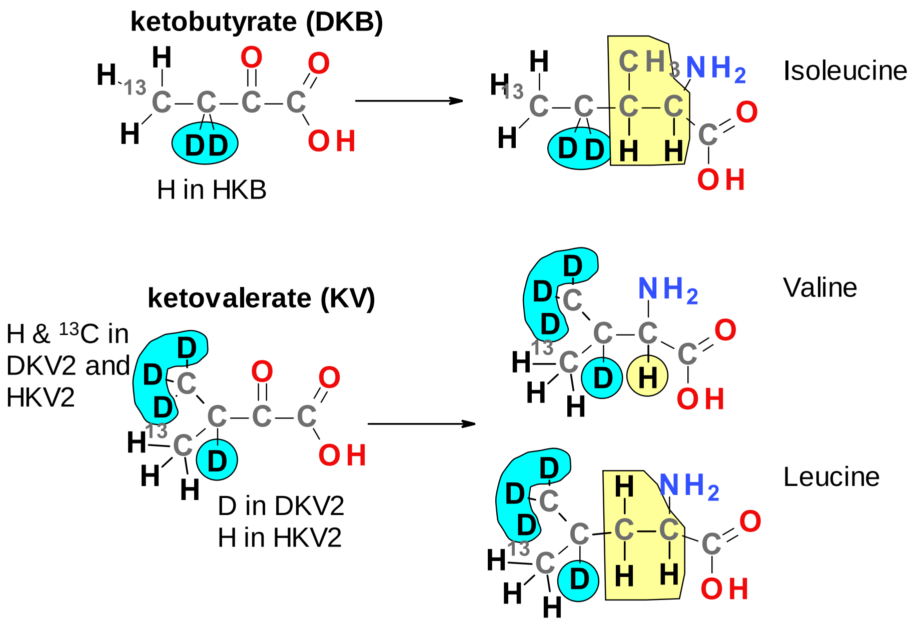

Scheme S1: Labeled Precursors and Isotope Distribution on Ile, Leu, and Val.

The deuterons highlighted in cyan on DKB and KV are replaced by protons in ketobutyrate to give HKB and in ketovalerate to give DKV2 (both methyl groups labeled, β-hydrogen deuterated) or HKV2 (both methyl groups labeled, β-hydrogen protonated). In the case of DKV2 and HKV2, the second protonated methyl would also be ^{13}C labeled. Proton content of the yellow highlighted group on the amino acids synthesized from the precursors will reflect the proton content in the growth media.

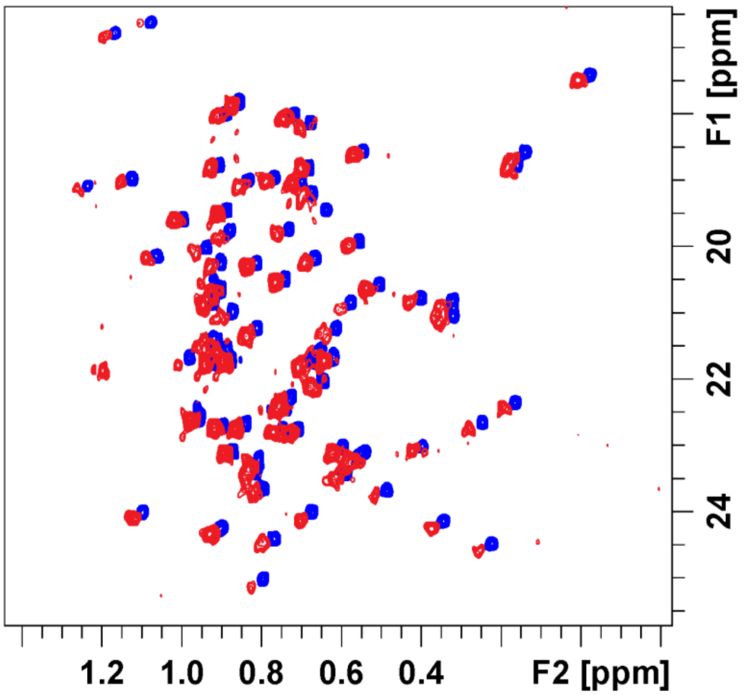

Figure S1: Overlay of Leu-Val region for 0% (red) and 90% (blue) D_2O. Both spectra were acquired under the same conditions (NS=8, 80 complex points in carbon).

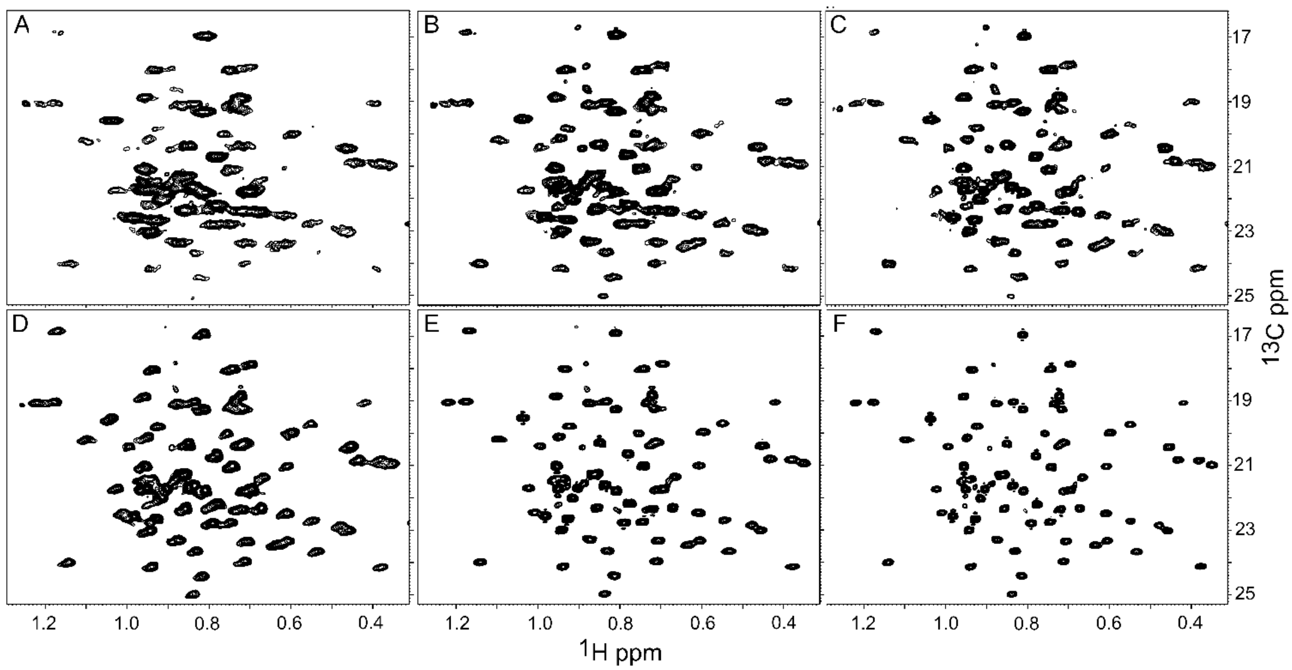

Figure S2: Effect of Solvent D_2O Level and Ketovalerate precursors on Quality of Leu, Val Spectrum. Cells were grown in H_2O+HKV2 (A), H_2O+DKV2(B), H_2O+KV (C), 90% D_20+HKV2 (D), 90% D_2O+DKV2 (E), 90% D_2O+KV (F). The quality of the spectra aligns with the MQ relaxation rates: 99 s^{-1} (A), 67 s^{-1} (B), 49 s^{-1} (C), 58 s^{-1} (D), 43 s^{-1} (E), 19 s^{-1} (F).

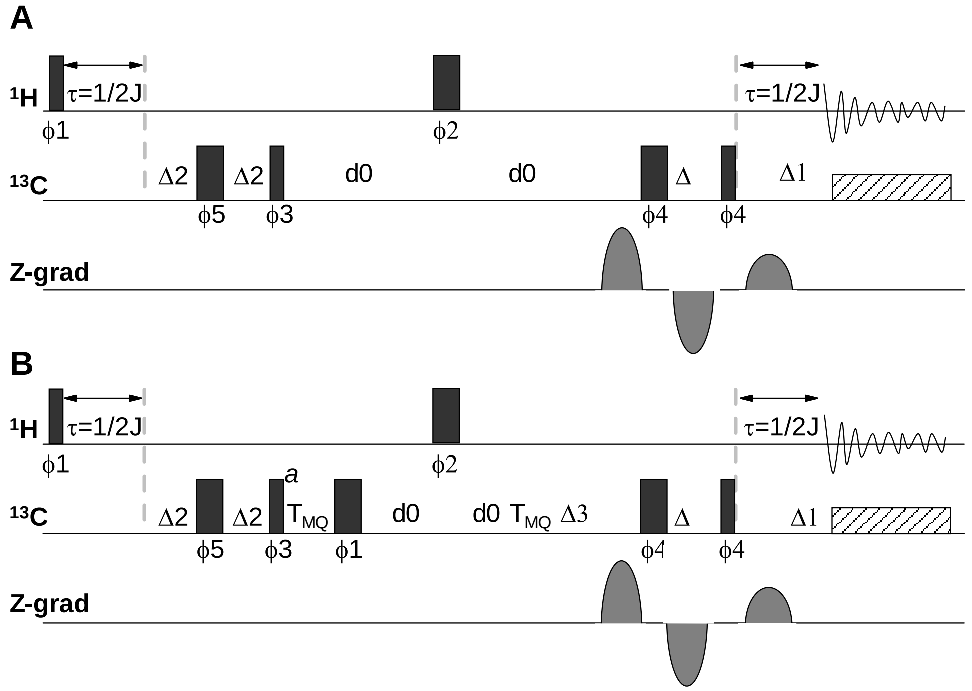

Figure S3: HMQC Pulse Sequences. Panel A shows the HMQC sequence used to acquire 2D-^{1}H-^{13}C correlated spectra, panel B shows the sequence used to measure MQ relaxation. Narrow rectangles are 90° pulses (^{1}H ~13 μs, ^{13}C ~9.5μs), wide rectangles are 180° pulses. ^{13}C decoupling was accomplished using 70 μs 90° pulses with a garp decoupling scheme (crosshatched). Proton-carbon coupling constant (J) was 125 Hz. Coherence selection is accomplished by the three gradients, applied for 1 ms each in a ratio of 80:80:40.2, with a 200 μs recovery period. Delays Δ, Δ2, and Δ3 ensure that the proton evolution time is equal on both sides of the central proton 180° pulse, e.g. Δ3=^{13}C 180°.Carbon evolution is obtained by incrementing d0. Magnetization at point *a* (panel B) is multiple quantum (MQ) (I_xC_y). MQ relaxation rate was obtained by increasing T_{MQ} and measuring the decay of the signal. Phases were: ϕ1: x, ϕ2: x x -x -x, ϕ3: x -x, ϕ4: x x x x -x -x -x -x, ϕ5: x, ϕ_{rec}: x -x x -x -x x -x x.

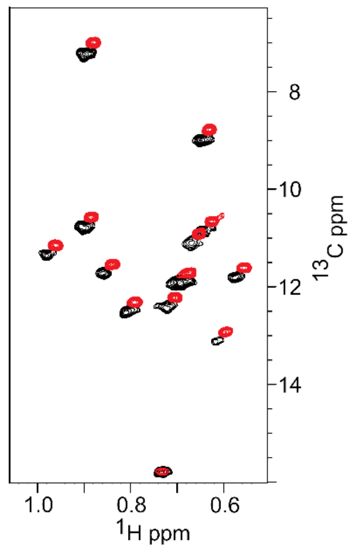

Figure S4. Deuterium Isotope Shifts. An overlay of the Ile region for a sample grown on protonated ketobutyrate (HKB, black) or deuterated ketobutyrate (DKB, red). Both samples were grown in KV and 90% D_2O, 90% ^{2}H-glucose. The upper part of the panel (< 14 ppm ^{13}C) shows Ile resonances. The peak at the very bottom of the panel is from a Leu residue, showing no deuterium isotope shift between the samples, as expected.

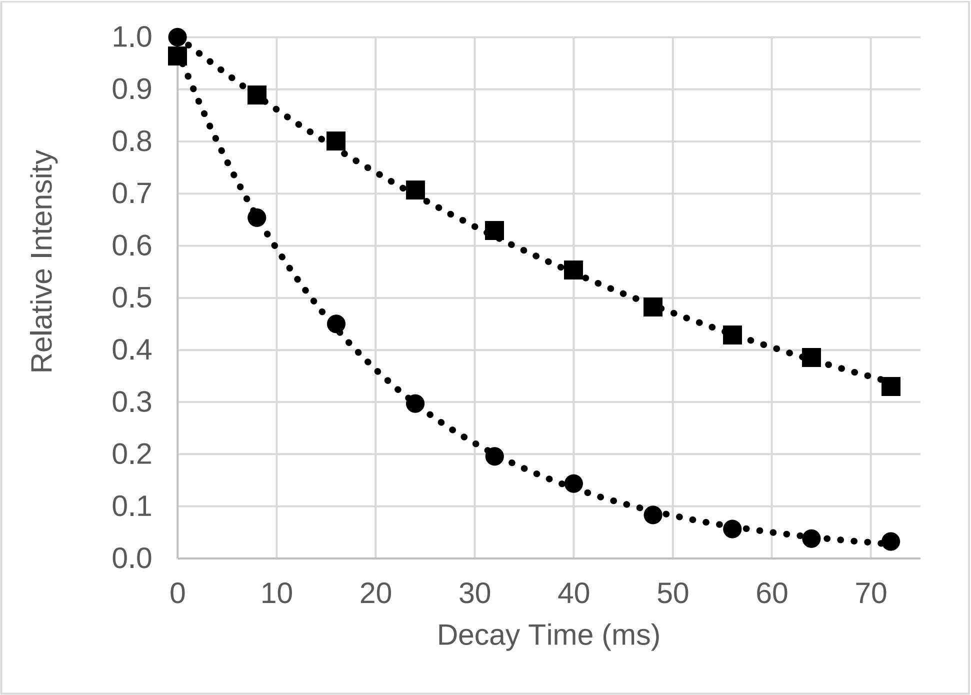

Figure S5: Decay of the ^{15}N α and β spin states from 1D-TRACT spectra.
